# Editorial: Immune-related adverse events for patients with lung cancer-volume II

**DOI:** 10.3389/fonc.2023.1282394

**Published:** 2023-11-08

**Authors:** Yusha Wang, Xia Wang, Cheng Zhan, Benjamin Frey, Udo S. Gaipl, Hubing Shi, Xuelei Ma

**Affiliations:** ^1^ Department of Biotherapy, Cancer Center and State Key Laboratory of Biotherapy, West China Hospital, Sichuan University, Chengdu, Sichuan, China; ^2^ Department of Biotherapy Research, West China Hospital, Sichuan University, Chengdu, Sichuan, China; ^3^ Department of Thoracic Surgery, Zhongshan Hospital, Fudan University, Shanghai, China; ^4^ Department of Radiation Oncology, Universitätsklinikum Erlangen, Erlangen, Germany

**Keywords:** immune-related adverse events (irAEs), immune checkpoint inhibitors (ICIs), Immunotherapy, lung cancer, editorial

Lung cancer, recognized for its high morbidity and poor prognosis, has been established as a common public health challenge worldwide. In recent years, immunotherapies targeting immune checkpoints, such as programmed cell death-1 (PD-1), programmed cell death ligand (PD-L1), and cytotoxic T-lymphocyte-associated antigen 4 (CTLA-4), have revolutionized therapeutic modalities in lung cancer. However, while immune checkpoint inhibitors (ICIs) exert substantial efficacy in patients with lung cancer, immune-related adverse events (irAEs) occur in different systems and organs owing to the overactivation of the immune system. Hence, integrated management of irAEs in lung cancer, including prevention, diagnosis, and treatment, is necessary. This Research Topic includes studies on the pathogenesis, diagnosis, and treatment of irAEs in patients with lung cancer. Importantly, this Research Topic brings together five papers from different perspectives showcasing advances in the management of irAEs in immunotherapy for lung cancer.

Clinical studies have revealed that ICI-induced pneumonitis, namely checkpoint inhibitor-induced pneumonitis (CIP), is the leading cause of fatal irAEs in ICI-treated patients. Therefore, it is important to clarify the potential mechanism, establish a standard diagnosis, and explore effective treatments for CIP in patients with lung cancer undergoing immunotherapy. Guo et al. comprehensively reviewed auxiliary methods and innovative possibilities for artificial intelligence-assisted CIP diagnosis of non-small cell lung cancer (NSCLC). Additionally, the authors discussed pathogenic mechanisms of T-cell subpopulation disorders, management of patients with CIP at all levels, and potential therapeutic strategies targeting cytokines and signaling pathways. Considering severe CIP, Gao et al. summarized the computed tomography characteristics, proposed bronchoalveolar lavage metagenomic next-generation sequencing, and discussed the feasibility of a noninvasive ventilation strategy in the prone position. Specifically, the authors proposed an in-depth mechanistic exploration and corresponding clinical concepts for CIP, from diagnosis to treatment. Furthermore, they presented the necessity, feasibility, and other valuable insights into the clinical management of CIP.

It is well-established that ICI-induced side effects are rarely observed in organs other than the immune system. Regarding immune-related skin toxicity, cutaneous adverse events include various inflammatory reactions, and CPI-associated bullous pemphigoid (BP) is less frequent. Wang et al. systematically reported cases of pembrolizumab-induced BP, known to be similar to classical BP. The authors suggested that treatment should include steroids, steroids combined with other drugs, and discontinuation of pembrolizumab therapy, depending on the severity and response. In addition, ICI-induced hematologic irAEs are rarer than those caused by chemotherapy. In the reported case, severe pancytopenia induced by the PD-1 antibody tislelizumab responded well to steroids; however, the patient developed cerebral infarction after platelet reduction (Gu et al.). These findings suggest that early identification of hematologic irAEs and assessment of cerebrovascular accident risk in patients with thrombocytopenia are critical for reducing potential adverse events.

Neurological irAEs, including encephalitis, aseptic meningitis, Guillain-Barré syndrome (GBS), and myasthenia gravis, have been associated with autoantibodies that target the nervous system. However, the manifestation, treatment, and prognosis of ICI-induced neurological toxicities remain poorly understood. Hence, a new regimen for ICI-induced GBS is discussed in the presented case report. The authors reported a patient with refractory ICI-induced GBS. Although routine treatments, including methylprednisolone and γ-globulin, were ineffective, the patient responded well to mycophenolate mofetil (Ding et al.). Collectively, patients with irAEs may benefit from immunotherapy; however, serious irAEs should be avoided. Although the incidence of irAEs is low, they are often dangerous or even fatal. Based on the standard management for rare irAEs, individuals who fail to respond appropriately to conventional treatments warrant special consideration, accompanied by measures to improve existing management processes and establish standard and comprehensive treatment measures.

In conclusion, this Research Topic describes cases that provide a basis for the management of irAEs in patients with lung cancer. Among these reports, Gao et al. reviewed a case of penpulimab, a PD-1 monoclonal antibody-induced CIP, and provided clinical insights for CIP diagnosis and treatment. The clinical features of pembrolizumab-induced BP were systematically reviewed, and the diagnostic and treatment steps for rare cutaneous side reactions were explored by Wang et al. Nevertheless, life-threatening and refractory pancytopenia caused by the PD-1 antibody tislelizumab was reported by Ding et al., highlighting the importance of early recognition of hematologic irAEs to reduce the potential risk of side effects. GBS induced by chemotherapy in combination with KN406, a PD-L1/CTLA-4 bispecific antibody, was detected in a patient with lung cancer, and an unconventional but feasible treatment option, i.e., mycophenolate mofetil, was discussed.

Overall, this series presents novel therapeutic strategies and provides insights into the management of irAEs. By focusing on clinically based evidence, this series aimed to improve the outcomes of patients with irAEs and seek immunotherapeutic approaches for lung cancer.

## Author contributions

YW: Data curation, Writing – original draft. XW: Writing – original draft. CZ: Writing – review & editing. BF: Writing – review & editing. UG: Writing – review & editing. HS: Writing – review & editing. XM: Writing – review & editing.

